# A low-cost portable system for 3-Axis measurement of static and extremely low frequency magnetic fields

**DOI:** 10.1016/j.ohx.2025.e00683

**Published:** 2025-07-18

**Authors:** Veronika Wohlmuthova, Michal Labuda, Mariana Benova

**Affiliations:** University of Zilina, Faculty of Electrical Engineering and Information Technology, Department of Electromagnetic and Biomedical Engineering, Univerzitna 8215/1, Zilina 010 26, Slovakia

**Keywords:** Electromagnetic fields, Magnetic field meter, ELF magnetic fields, Static magnetic fields, 3-axis magnetic sensing

## Abstract

Magnetic fields play a crucial role in modern science and technology - yet precise and accessible tools for their measurement remain limited, especially for small laboratories, educators, or independent researchers. This paper introduces a novel, open-source magnetic field measurement system based on three-axis sensors for monitoring both direct and extremely low frequency magnetic fields. The device features a modular hardware design centered around a custom PCB, enabling flexible analog filtering, Bluetooth data transmission, and offline LCD visualization. By combining the MC858 and MPU9250 sensors with precise analog signal conditioning and a 12-bit ADC, the system ensures reliable detection of magnetic fields including the 50 Hz mains frequency and its harmonics. To verify the functionality of the device, experimental measurements were conducted inside a Faraday cage using a common hair dryer placed at distances of 1 cm and 3 cm from the sensors as a source of electromagnetic field. Frequency analysis confirmed reliable detection of the dominant 50 Hz component and its harmonics, as well as the system’s ability to distinguish changes in field intensity based on distance and operating state of the source device.

## Graphical abstract

**Figure:**
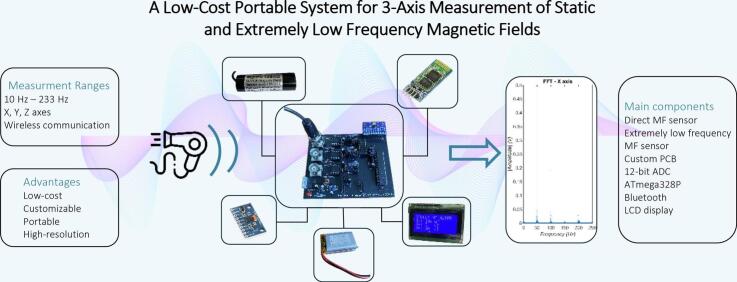

## Specifications table

Hardware name: A Low-Cost Portable System for 3-Axis Measurement of Static and Extremely Low Frequency Magnetic Fields
Subject area: Educational tools and open source alternatives to existing infrastructure
Hardware type: Field measurements and sensors
Closest commercial analog: Electromagnetic Field Meter ELT-400
Open source license: CC BY 4.0
Cost of hardware: 330 €
Source file repository: *https://doi.org/10.17632/4pjr32n2zm.1*

## 1 Hardware in context

Magnetic fields are an inseparable part of everyday life. They can be found in nature as the Earth's geomagnetic field, but also in technical devices through the low frequency fields generated by electrical equipment to high-voltage lines [1,2]. Due to the progressive electronification of society and the constant technological development, the human population is being overexposed to these fields. In recent years, this has led to an increased interest of the professional community in research on the potential biological effects of electromagnetic fields [3].

**Fig. 1:**
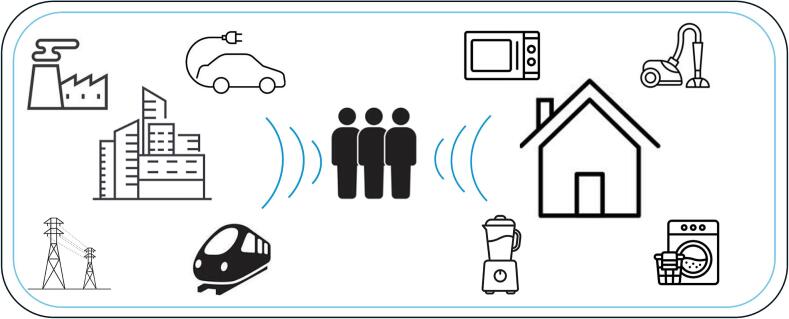
Electrical devices and equipment impacting the human population in the home or work environment.

Mains-powered devices typically emit magnetic fields at 50 Hz or 60 Hz, depending on country. It is these fields that are found in a typical home, school or work environment. Different sources of magnetic fields can be seen in Fig. 1. They are mainly generated by electrical appliances, electric motors, welding machines or power transmission cables. The fields generated by these devices fall into the so-called extremely low frequency fields (ELF, ≤300 Hz) whose effects have been intensively studied. Several studies have shown a possible correlation between long-term exposure to ELF fields and the incidence of childhood leukaemia [4,5], as well as with some neurodegenerative diseases. There has also been a lot of other research on memory disorders, headaches and sleep disorders related to ELF [2].

On the basis of these findings, international standards and recommendations have been developed setting acceptable levels of exposure. The International Commission on Non-Ionizing Radiation Protection (ICNIRP) has set a magnetic induction limit for 50 Hz at 200 μT for the general public [6]. The International Agency for Research on Cancer (IARC) has classified ELF magnetic fields as Group 2B - a possible human carcinogen [7].

The aim of magnetic fields research is therefore not only technological optimization but also the protection of public health.

Although most research relies on the use of professional measuring equipment, its higher cost and limited modification options present a limitation in some research contexts. While these devices provide high precision and validated calibration, they tend to be very limited in terms of their ability to be modified, customised for data collection or integrated with other systems. Among the best known commercially available solutions for the measurement of low frequency magnetic fields are devices from Narda Safety Test Solutions GmbH (Pfullingen, Germany), namely the EHP-50F [8] and ELT-400 [9] models. Both devices enable triaxial measurement of magnetic fields over a wide frequency range from 1 Hz to 400 kHz, Other commercial devices include the NFA30M [10] with a measurement range of 16–32 kHz from Gigahertz Solutions (Langenzenn, Germany) and the EMDEX II [11] (EMDEX LLC, Patterson, California) with a range of 40–800 Hz. Other instrument models from different manufacturers are also available on the market covering the full spectrum of ELF magnetic fields - typically in the range 1 Hz to 300 Hz.

The main limitations of existing commercial equipment for use in scientific research are the high purchase price and the limited possibilities for customisation of hardware and software components. In many cases it is not possible, or is technically and financially very difficult, to adapt these devices to specific research requirements. To overcome these barriers, while promoting wider awareness and accessibility of magnetic field measurements, we have designed a low-cost, wireless and modular device for measuring static and time-varying magnetic fields in the 7–230 Hz range in this work. The proposed solution provides full control over data acquisition, processing and analysis, making it a suitable tool for flexible and independent research.

The main advantages of the device include its open architecture, which allows easy expansion with additional sensors according to the needs of a specific application. This feature fundamentally increases the device's applicability in a wide range of scientific and technical fields, including environmental monitoring, biomedical research or teaching in the field of electromagnetic compatibility.

## 2 Hardware description

### 2.1 Measuring setup

The aim of this paper is to present the development of a low-cost device designed to sense both static and time-varying magnetic fields in three axes.

The main requirements in the design of the device were low acquisition cost, accessibility to the general public and ease of assembly. Another key requirement was the ability to flexibly adjust the parameters of the analog filters without the need for physical intervention in the hardware. Last but not least, emphasis was placed on compactness, portability and easy handling of the equipment.

The hardware part of the device consists of several components, with a custom printed circuit board (PCB) at its core, designed for proper signal processing and transmission. The PCB contains a connectors for the alternating (AC) and direct (DC) magnetic field sensors, protection elements, operational amplifiers (OAs), analog-to-digital converter (ADC), a microcontroller unit (MCU) and a Bluetooth module. The device also allows the connection of an LCD display for offline visualization of measured static magnetic field induction values in three axes.

A cylindrical-shaped MC858 [12] sensor (Magnetic Sciences Inc., Acton, USA), with a frequency range of 10 Hz–400 Hz and a maximum response at a frequency of approximately 58 Hz, was chosen to sense the alternating magnetic field in the desired range. This range allows reliable measurement of 50 Hz signals as well as detection of higher harmonic frequencies. The output of the sensor is in the form of an induced voltage in millivolts (mV), corresponding to the magnetic field strength in mT. The frequency range of the output voltage corresponds to the frequency spectrum of the sensed field. Since the MC858 sensor senses the magnetic field in only one axis, three sensors, one for each axis (X, Y and Z), must be used for a three-axis measurement.

The sensitivity of the sensor is 22 mV/μT and it has the highest response to a magnetic field oriented parallel to its axis - in the case of the MC858 this is the longitudinal axis of the cylinder. The sensor is calibrated and certified to ANSI 644-1987 [12].

The MPU9250 [13] sensor (TDK InvenSense, San Jose, USA), which integrates a three-axis magnetometer with a range of ± 4800 μT, operating on the Hall effect principle, was used to sense the static magnetic field.

The output data from the MPU9250 sensor is displayed on the attached LCD display - I2C Serial Interface 1604 LCD Module [14], (Handson Technology, Shenzhen, China). For the purpose of possible device customization and sensor replacement, a BNC connector, in our case type B6251F1-NT3G-50 (Amphenol RF, Danbury, USA), has been integrated on the PCB.

To protect the input circuits, protecting diodes, specifically model 1 N4148 [15] (Microchip Technology, Chandler, USA), were included at the input of the OA to limit the input voltage to a power supply range of 0 V–5 V and thus prevent damage to the electronic circuit.

The signal is then fed to the voltage follower represented by OA MCP602-I/P [16] (Microchip Technology, Chandler, USA), with a separate OA integrated circuit dedicated to each sensor. The voltage followers were used for impedance decoupling between the sensors and the rest of the analog circuit. This type of OA was chosen because of its high-speed operation, low power consumption, wide supply voltage range, rail-to-rail output swing, low input bias current and low voltage offset, which makes it an ideal choice for processing low-amplitude signals from magnetic sensors.

Given that the device is designed to detect the mains frequency of 50 Hz and its harmonics, analog filters were implemented to ensure selective amplification of those frequency components and suppression of interference.

**Fig. 2:**
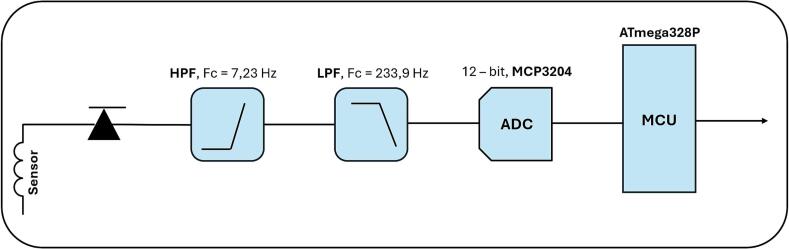
Block diagram of the analog section of the device.

The active high-pass frequency (HPF) filter for each channel (axis) is formed by a series connection of a capacitor C_1_; C_2_; C_3_ with a capacitance of 100 nF and a resistor R_1_; R_2_; R_3_ with a resistance of 100 kΩ, the resulting cutoff frequency of the filter being approximately 7.23 Hz, Fig. 2, Fig. 6.

An active low-pass frequency (LPF) filter is also implemented in this section, which consists of a parallel connection of a capacitor C with a capacitance of 6.8 nF and a resistor R with a value of 100 kΩ. The resulting cutoff frequency of this filter is approximately 233.9 Hz, which allows the higher harmonic frequencies of the signal to be captured, Fig. 2. The signal is also in same stage amplified by inverting OA with a total gain of −1. The total gain can be adjusted by changing the value of resistor R. LPF filter cutoff frequency can be also adjusted by changing the value of capacitor C, Fig. 6.

The signal is then digitized using a 12-bit ADC MCP3204-CI/P [17] (Microchip Technology, Chandler, USA), at a sampling rate of 500 Hz, Fig. 2. Compared to commonly used 10-bit converters (e.g. the integrated ADC in the ATmega328P MCU), the MCP3204 provides higher resolution, small quantisation step and therefore more accurate low-amplitude voltage measurement.

The converter allows sampling of up to four independent analog inputs, enabling simultaneous measurement of signals from all three magnetic sensors without the need for an additional multiplexer. In addition to the technical advantages, its low cost was also an important criterion, contributing to the overall low cost of the device.

The digitized data is then processed by an 8-bit ATmega328P [18] MCU (Microchip Technology, Chandler, USA). The ATmega328P is a popular and frequently used MCU with its low cost and high availability. Its ease of programmability and wide support in communities also allows easy customization and extension of our device functionality.

The Bluetooth module HC-06 [19] (HC Electronics, Shenzhen, China) was used for wireless communication with the computer, with a baudrate set to 38,400 Baud. The module acts as virtual serial port and provides reliable real-time data transmission and is compatible with most commonly available Bluetooth receivers.

The device is powered by two series-connected rechargeable lithium-polymer (LI-Pol) batteries LP803450 (Cellevia Batteries, Łódź, Poland) with a nominal voltage 3.7 V of each and capacity 1500 mAh of each [20]. Batteries output is connected to the low dropout 5 V voltage regulator LM2940-5.0 [21] (Texas Instruments, Dallas, USA). This regulator has the ability to source 1 A of output current with a dropout voltage typically 0.5 V.

**Fig. 3:**
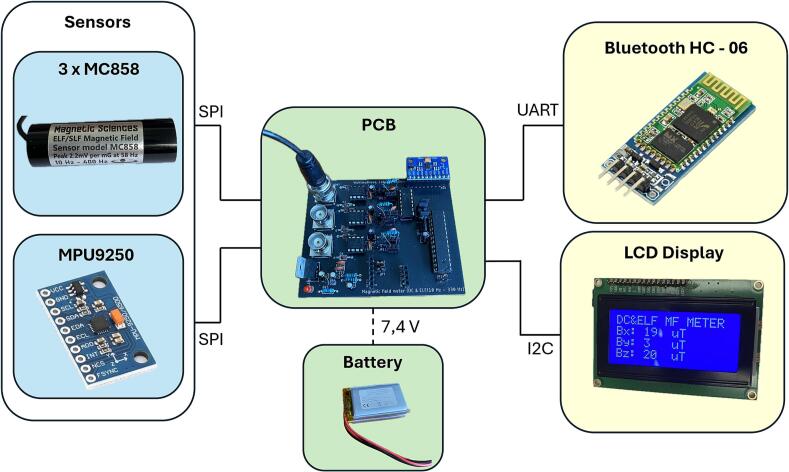
Functional diagram of the proposed measurement system and its communication interfaces.

This arrangement makes it possible to obtain sufficient voltage to power all components while maintaining the compact size and good battery endurance in real conditions. The whole measurement system can be seen in Fig. 3.

• The device is affordable, enabling low-cost deployment in both laboratory and field settings.
• The device is customizable, allowing users to easily modify filtering parameters or integrate alternative sensors.
• The device has the potential to expand knowledge of magnetic fields by enabling detailed, real-time measurements across different environments

### 2.2 Li-Pol battery charging circuit

A simple charging circuit was designed to provide charging of both Li-Pol batteries, with the option of connecting via a USB connector. The files needed for the construction can be found under the name Charging circuit.

The integrated circuit MCP73831T-2ATI/OT [22] (Microchip Technology, Chandler, USA), was used to control the charging process. The circuit is powered by a standard 5 V supply through a USB port. Capacitor C_1_ is connected at the input to filter interference from the power supply, while capacitor C_2_ is used to stabilize the output voltage.

The value of the charging current is determined by the resistor R_1_, connected to the PROG pin - in this case a resistor with a value of 2 kΩ was chosen to define the maximum charging current 500 mA.

**Fig. 4:**
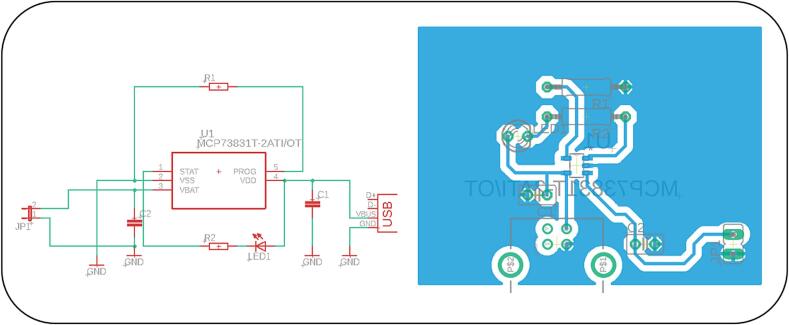
Electronic circuit and PCB layout of the charging device designed in Autodesk Eagle.

The charging status is indicated by an LED (LED1) connected to the STAT pin (pin 1), Fig. 4. During active charging, the LED is on, and when the battery is fully charged or not connected, the LED goes off.

This type of circuit is ideal for simple, compact projects where reliable charging of Li-Pol batteries from a USB source is required. The main advantages include not only its small size and low power consumption, but also the minimal number of external components required, making it easy to integrate into a variety of devices.

**Fig. 5:**
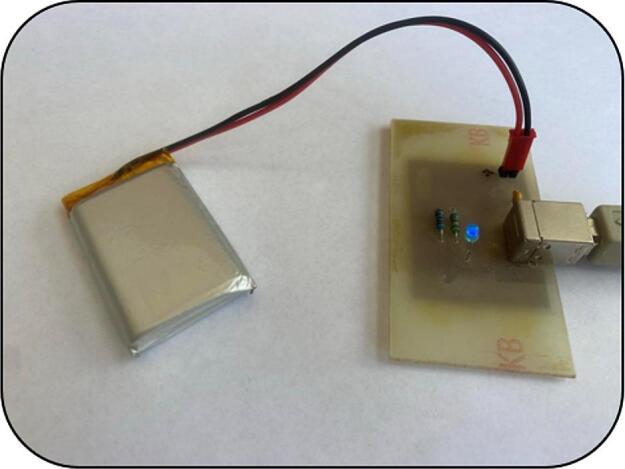
Connection of Li-Po battery for the charging process using our charging circuit**.**

Note: Charging can only be done one battery at a time, Fig. 5. When connecting to the charger, it is important to correctly connect the positive terminal of the battery to the corresponding jumper marked with the symbol ‘+’ to prevent damage to the battery or charging module.

## 3 Design files summary

**Table:** 

**Design file name**	**File type**	**Open source license**	**Location of the file**
ELF_meter_PCB	CAD Files	CC BY 4.0	*https://doi.org/10.17632/4pjr32n2zm.1*
DC_ELF_Meter_firmware	Microchip studio Files	CC BY 4.0	https://doi.org/10.17632/4pjr32n2zm.1
Charging circuit	CAD Files	CC BY 4.0	https://doi.org/10.17632/4pjr32n2zm.1
Protective_cover.stl	CAD Files	CC BY 4.0	https://doi.org/10.17632/4pjr32n2zm.1
Magnetic_field_meter_app.m	Matlab Files	CC BY 4.0	https://doi.org/10.17632/4pjr32n2zm.1
Magnetic_induction_MC858.m	Matlab Files	CC BY 4.0	https://doi.org/10.17632/4pjr32n2zm.1

## 4 Bill of materials summary

**Table:** 

**Designator**	**Component**	**Number**	**Cost per unit −currency**	**Total cost −** **currency**	**Source of materials**
PCB	PCB	5	0.4 €	2 €	https://jlcpcb.com/
D1 – D6	Protecting diode 1 N4148	6	0.10 €	0.60 €	https://shorturl.at/9Z7Wm
R, R1 – R3, R7, R8	Resistor 100 kΩ	8	0.13 €	1.04 €	https://shorturl.at/MEgE0
R4	Resistor 470 Ω	1	0.50 €	0.50 €	https://shorturl.at/2JrAg
C1 – C3	Capacitor 220 nF	3	0.43 €	1.29 €	https://shorturl.at/sB5Ea
C	Capacitor 6,8 nF	3	0.41 €	1.23 €	https://shorturl.at/Vt9b2
C7, C11, C12	Capacitor 10uF	3	0.10 €	0.30 €	https://shorturl.at/vEqtZ
C8, C10	Capacitor 100nF	2	0.67 €	1.34 €	https://shorturl.at/GcPwc
C9	Capacitor 4,7 uF	1	0.57 €	0.57 €	https://shorturl.at/UhgfY
J1,J2,J3,J4,J14,J15, J16,J17	Male pin header	1	1.70 €	1.70 €	https://shorturl.at/XCDsx
J8–J13, HC05, LCD display, MPU −9250	Female pin header	3	2.54 €	7.62 €	https://shorturl.at/lLUIq
MCP602P 8 dip socket	PDIP-8	3	0.99 €	2.97 €	https://shorturl.at/DRru5
A/D converter 14 dip socket	PDIP-14	1	0.22 €	0.22 €	https://shorturl.at/q7eww
ATmega328P 28 dip socket	PDIP-28	1	0.23 €	0.23 €	https://shorturl.at/giIiR
J1, J2, J3, J4, J14, J15, J16, J17	Jumper (Shunt)	7	0.38 €	2.66 €	https://shorturl.at/dmq8I
BNC Conector	Coaxial cable connector for MC858	3	7.38 €	22.14 €	https://shorturl.at/kWgHx
U3 Voltage regulator	Fixed voltage regulator – 5 V	1	1.68 €	1.68 €	https://shorturl.at/X5sOE
LED1	LED diode	1	0.18 €	0.18 €	https://shorturl.at/n5xEY
MPU-9250	3-axis static magnetic field sensor	1	14.59 €	14.59 €	https://shorturl.at/BqyMl
Bluetooth Modul HC-06	Bluetooth Module	1	6.63 €	6.63 €	https://shorturl.at/hzzY8
LCD	LCD Display	1	6.5 €	6.5 €	https://shorturl.at/zdgyM
MC858	AC magnetic field sensor	3	83.62 €	250.89 €	https://shorturl.at/kVwMg
3D printer filament	PLA Matte	1	23.76 €	5.36 €	https://shorturl.at/vCtcH
**Total cost:**	332.24 €				

*All prices are exclusive of VAT and applicable duty charges.

## 5 Build instructions

The assembly of the device itself consists of two stages:

• Assembling and connection of electronic components - includes assembling the PCB with all the necessary electronic components, connecting the LCD display, preparing the power supply system and basic visual check of the reliability of the connections.
• Control system implementation and device completion - includes uploading functional software to the ATmega328P MCU via a suitable programmer. After successful installation, the device needs to be placed in a protective box that ensures mechanical stability and protection from external influences.

### 5.1 Other necessary tools for assembly

• Soldering iron (50–70 W)
• Solder wire (Sn60Pb40 / lead-free)
• Multimeter
• Side cutters
• Atmel ICE Programmer (for ATmega328P) [23]

### 5.2 PCB assembly

For PCB manufacturing, we recommend using the services of one of the established manufacturers who can deliver high quality and affordable PCBs based on the Gerber files. For this project, we have chosen a reliable supplier JLCPCB who guarantees low cost, high precision and quality manufacturing.

**Fig. 6:**
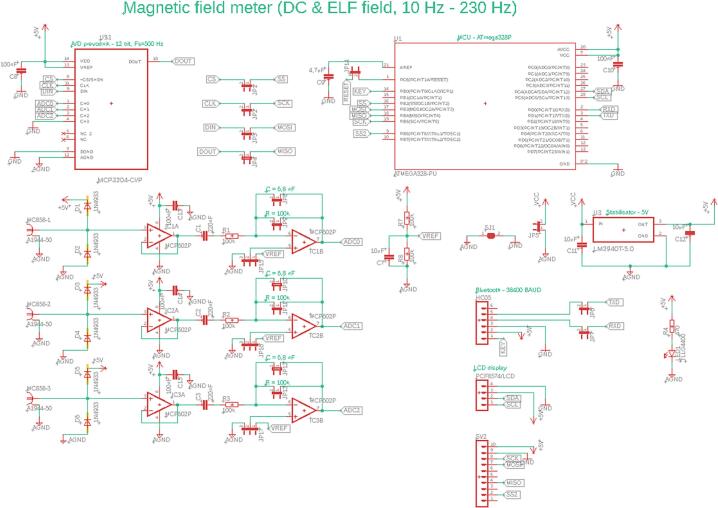
Device schematic created in EAGLE CAD Software.

The first practical step in assembly is to fit the manufactured PCB board with all the electronic components as shown in the schematic. Fig. 6. illustrates the circuit diagram created in Autodesk Eagle design software. Which can be found under the name in our uploaded files: ELF_meter_PCB.

**Fig. 7:**
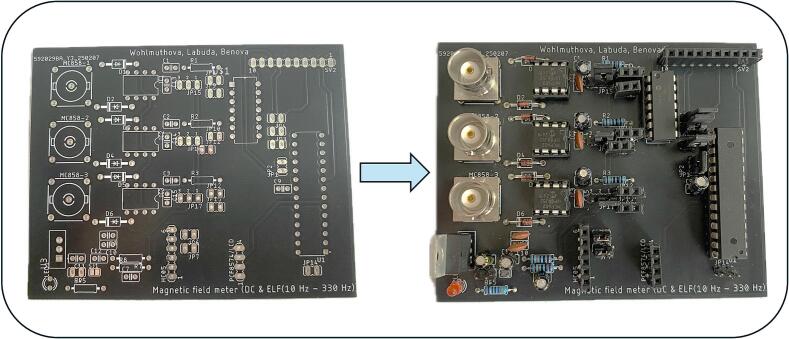
PCB board before and after components assembly.

For high-precision and time-sensitive applications, automated component placement and assembly services offered by PCB manufacturers can be utilized. However, this typically results in higher overall production costs. Within our design, the components are arranged on the board with clarity and accessibility in mind, which greatly simplifies manual assembly - even for less experienced users, like we can see in Fig. 7. To ensure correct and efficient mounting of the board, we recommend the following assembly order:

1. Low profile components - resistors and diodes.
2. Female pin headers - sockets and hollow connectors for OA, ADC and ATmega328P MCU.
3. Capacitors and other connector components – e. g. male pin headers.
4. High profile elements - BNC connectors.
5. Final modification - removal of overhanging components leads with side cutters.

**Fig. 8:**
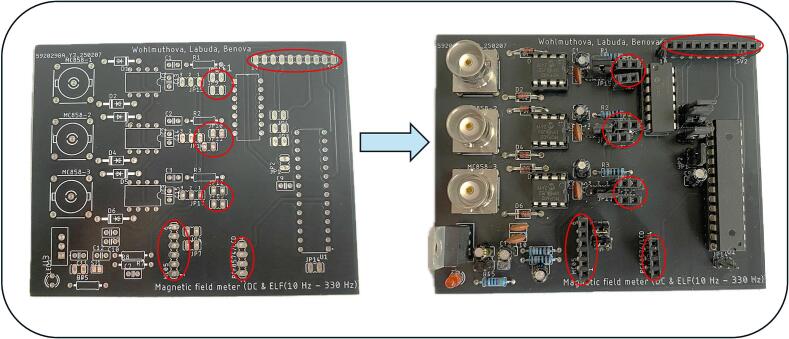
Positioning of female pin headers on PCB.

To ensure easy modifiability of the board, female pin headers were fitted in place of the LPF filters to allow quick replacement of passive components. In this way, it is possible to flexibly adjust the cutoff frequency of the filter without the need for mechanical intervention in the PCB board, Fig. 8. In the same way, DIP sockets and female pin headers were installed at the designated positions for the OAs, MCU, the LCD display and the Bluetooth module, which significantly simplifies the assembly and replacement of the individual parts of the device.

**Fig. 9:**
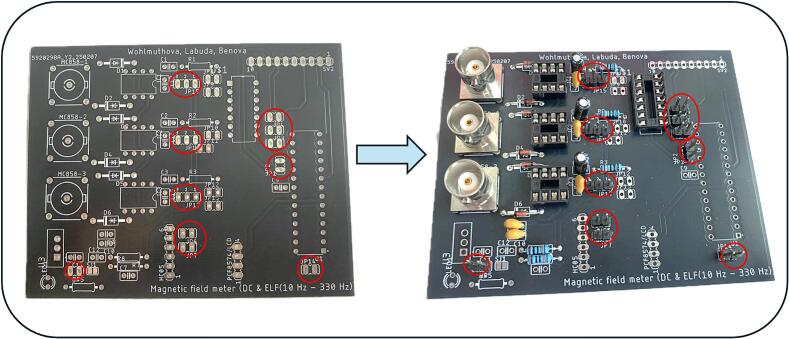
Positioning of male pin headers on PCB.

Description of individual jumpers (Fig. 9) and their functions:

JP6, JP7 - Enabling bidirectional communication between Bluetooth module and MCU.

JP1 – JP4, JP14 - for uploading the code to the MCU.

JP15 – JP17 - allows configuration of the DC offset of the OA. When the jumper is placed between pins 3 and 2, DC offset is set to 0 V. When the jumper is set between pins 2 and 1, to the OA's output is shifted by a 2.5 V reference voltage, effectively adding a 2.5 V DC offset.

**Fig. 10:**
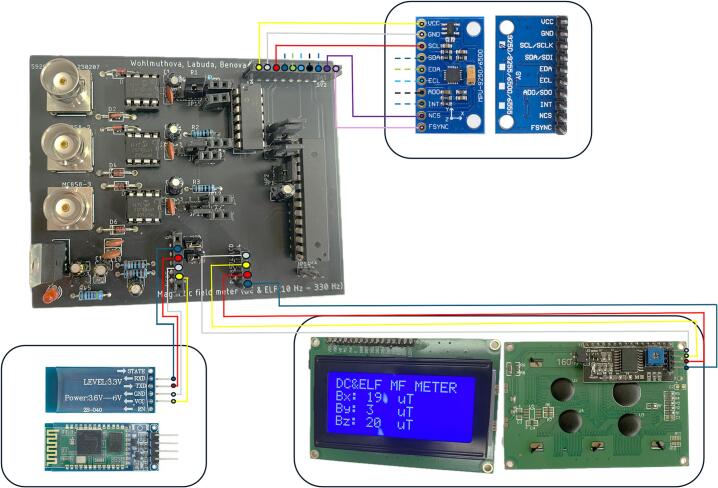
Block diagram illustrates the connections between the peripheral devices and the PCB.

After assembling the PCB with fixed components, peripheral components such as sensors, the Bluetooth module, and the LCD display are connected. Fig. 10. shows a block diagram illustrating the connection of individual peripheral components. To improve flexibility in positioning the display, we recommend connecting it via jump wires, which allows easier handling during final installation into the enclosure. The Bluetooth module and the MPU9250 sensor can be inserted directly into the female pin headers on the PCB.

If adjustment of individual component values is not required, the elements mounted in female pin headers may be permanently soldered to the PCB.

### 5.3 Preparation of batteries and power switch of the device

To ensure the functionality of our device, the device is powered by two Li-Pol batteries connected in series. These batteries are rechargeable through our custom charging circuit, allowing for reuse and reducing overall costs.

**Fig. 11:**
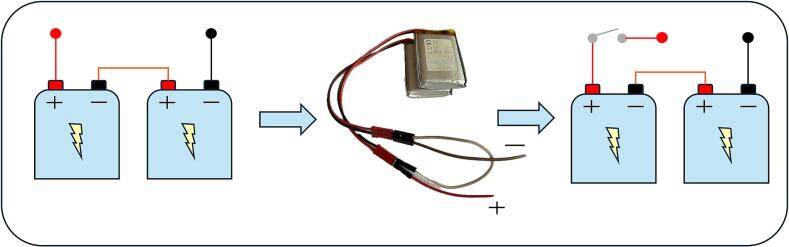
Series configuration of Li-Po batteries integrated with a power switch for efficient power management.

To enable automatic power on and off without manual intervention, a power switch has been integrated, Fig. 11, into the power circuit, connected to the positive terminal after the batteries.

Once all components are connected and power is supplied to the board, the red indicator LED located in the lower left corner of the PCB will light up, signalling the presence of power.

### 5.4 Uploading the firmware to the MCU

After assembling the printed circuit board and connecting all components, it is necessary to upload the functional firmware to the ATmega328P MCU. For this purpose, we used the freely available development environment Microchip Studio. This firmware can be found under the name: DC_ELF_Meter_firmware, in our uploaded files. This integrated development environment (IDE) is specifically designed for AVR and ARM MCUs developed by Microchip Technology. Microchip Studio provides tools for writing, debugging, and compiling code in C/C++, and enables direct uploading to the target MCU via a compatible programmer.

In our case, the Atmel-ICE Debugger was used as the programmer, with communication occurring through the ISP (In-System Programming) interface using SPI pins. The correct pinout for connecting the Atmel-ICE to PCB is shown in Table 1.

**Table 1:** Pinout for connecting the Atmel-ICE Debugger to PCB [18].

**Atmel-ICE AVR ports pins**	**Target pins**
Pin 1	SCK
Pin 2	GND
Pin 3	MISO
Pin 4	5 V
Pin 6	/RESET
Pin 9	MOSI

Steps for establishing the connection between the programmer and the PCB:

1. Remove jumpers and disconnect the Bluetooth module (see Fig. 12).

**Fig. 12:**
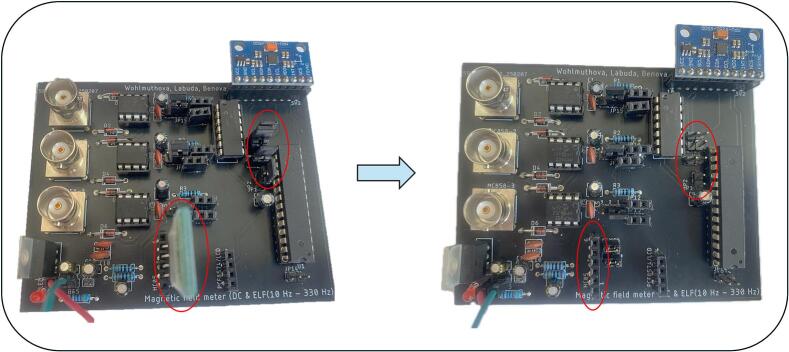
Removal of jumpers and Bluetooth module.
2. Connect individual programmer pins according to Table 1 and Fig. 13. (red, green, blue, yellow wires).

**Fig. 13:**
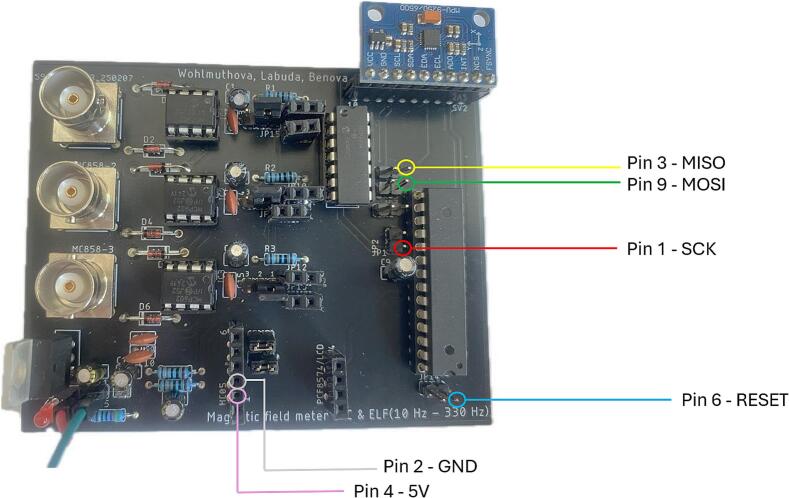
Visual representation of connection of Atmel-ICE Debugger to PCB*.*
3. Connect the Atmel-ICE programmer to the power supply (pink and white wires), Fig. 13.
4. Connect the power supply wires to the PCB.

Firmware upload procedure:

1. After connecting the programmer and supplying power, open the firmware project in Microchip Studio.
2. After launching Microchip Studio, click on the programming icon indicated in Fig. 14, Fig. 1.).

**Fig. 14:**
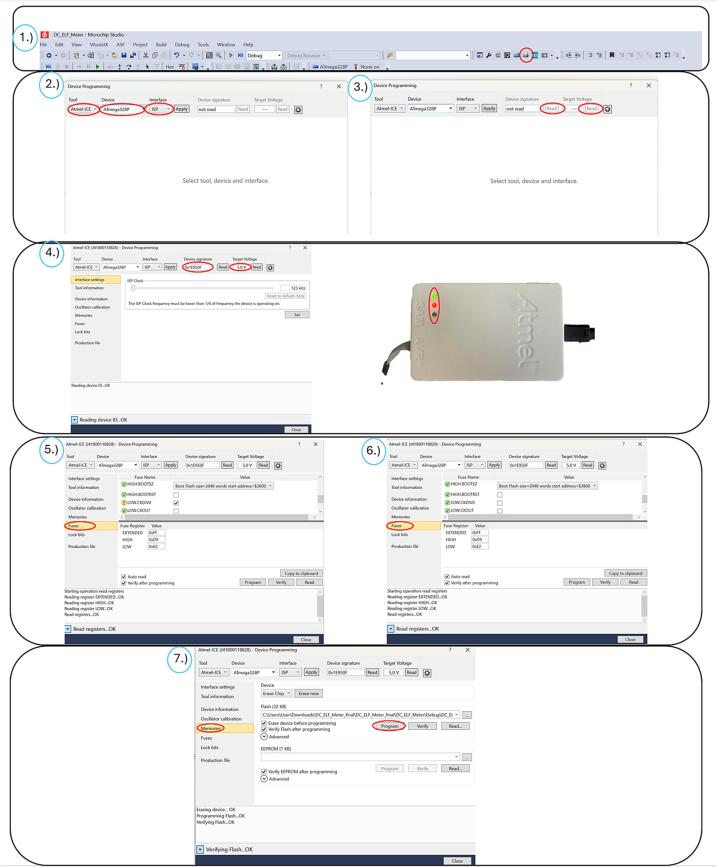
Step by step firmware upload procedure.
3. In the programming window, select Tool: Atmel-ICE, Device: ATmega328P, and Interface: ISP. Confirm the selection by clicking 'Apply', Fig. 14, Fig. 2.).
4. Read the Device Signature and Target Voltage, Fig. 14, Fig. 3.).
5. If the values are correctly detected, the programmer will signal the connection by lighting the green LED, Fig. 14, Fig. 4.).
6. For a new ATmega328P MCU, navigate to the Fuses tab and uncheck the LOW.CKDIV8 option. This option represents the default clock divider of 8, which limits the MCU’s frequency to 1 MHz. Disabling it sets the F_CPU frequency to 8 MHz, Fig. 14, Fig. 5.), 6.).
7. Navigate to the Memories tab, load the desired HEX file, and click on 'Program', Fig. 14, Fig. 7.).
8. After successfully uploading the code, the programmer can be disconnected, and the jumpers and Bluetooth module should be reconnected according to the schematic (see Fig. 12).

### 5.5 Design and print of the protective cover for the device

**Fig. 15:**
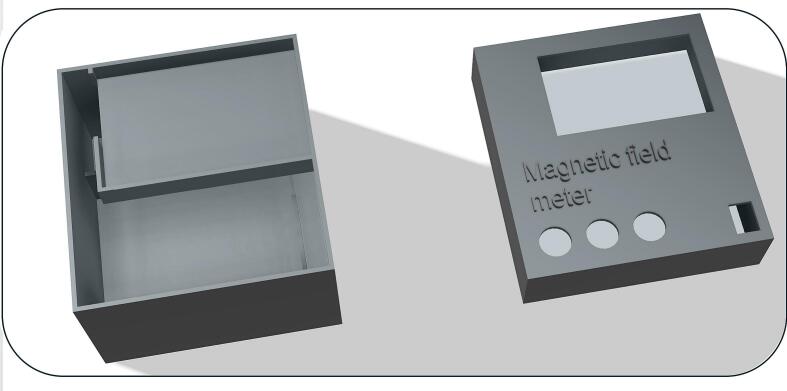
Protective enclosure designed in Autodesk Fusion 360.

In order to protect the device from external influences and mechanical disturbances, a protective box was designed in Autodesk Fusion 360 software, Fig. 15. The resulting model was then produced using 3D printing on a Bambu Lab A1 Mini printer. The box is used to safely store all the electronic components of the device, with the sensors themselves placed outside the protective cover to maintain flexibility in setting various measurement conditions.

For easy modification, the model remains available in.stl format (Protective_cover.stl), which can be easily edited in Autodesk Fusion 360 according to individual requirements.

The process of installing the device into the protective enclosure.

1. 3D print both parts of the protective enclosure using an available 3D printer.
2. After printing, remove all support structures.
3. Tape over all exposed contacts on the wires with suitable insulating tape.
4. Place the assembled device into the enclosure, following the schematic in Fig. 16.

**Fig. 16:**
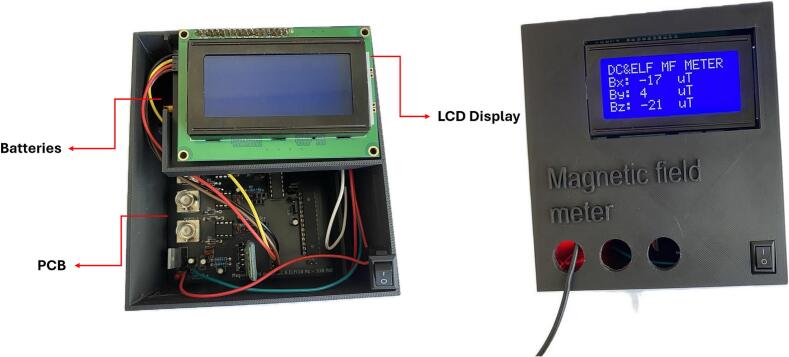
Placement of the device within the protective enclosure.
5. Secure the device's switch to the enclosure (e.g., using electrical tape).

## 6 Operation instructions

After successfully assembling the PCB and programming the ATmega328P MCU, data collection could begin. Once the device is powered on, the values of the magnetic induction of the static magnetic field along the X, Y, and Z axes are automatically displayed on the LCD screen after approximately 10 s (settling and calibration time after first start). These values are then updated at regular intervals every 2 s.

Communication between the device and the computer is implemented through the HC-06 Bluetooth module. For data visualization, processing, and archiving, a graphical application was developed using MATLAB App Designer Toolbox. You can find this application in the uploaded files under the name: Magnetic_field_meter_app.m. This approach enables not only the clear display of measured data but also their direct processing and analysis within the MATLAB environment.

To run the application, the user must have MATLAB installed and a functional Bluetooth module (internal or external). Once the device is powered on, the red LED on the Bluetooth module starts blinking, indicating its readiness for pairing. Pairing with the computer is initiated by entering the default password '1234′, with the default device name set as 'HC-06′.

After successful pairing, it is necessary to manually select the correct COM port in the application following the procedure outlined below:

1. After powering on the board and connecting to the PC via Bluetooth, open the Device Manager in Windows.
2. Navigate to the 'Ports (COM & LPT) ' section.
3. Identify the newly assigned COM port for the Bluetooth module.
4. Enter the obtained COM port number into the application's source code in the following format: app.s = serialport(“COM6″, 38400); (In this example, the COM port is COM6), Fig. 17.

**Fig. 17:**
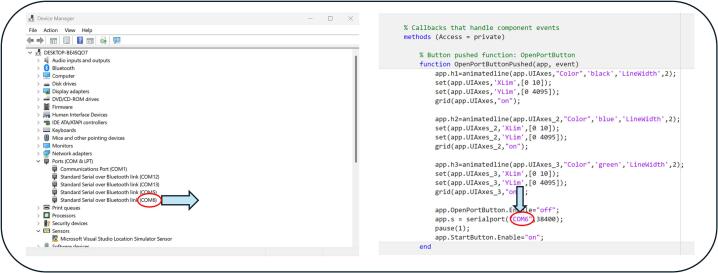
Identification of the newly assigned COM port and its implementation in the application's source code.
5. Save the changes made.

**Fig. 18:**
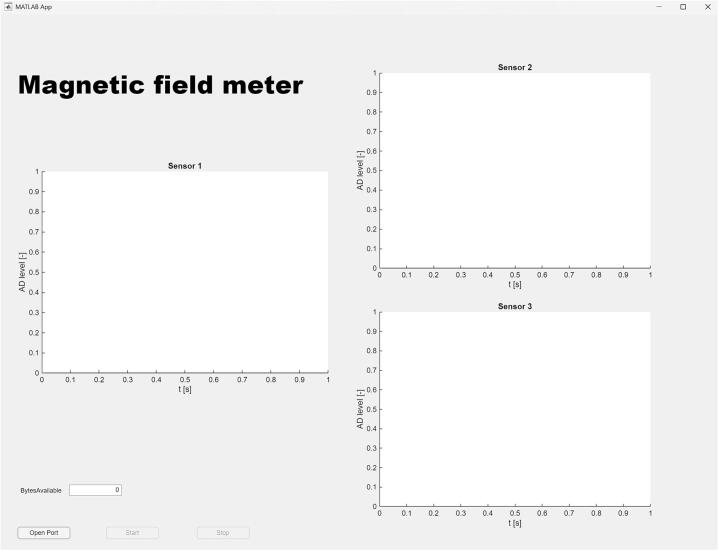
Graphical User Interface of the MATLAB Application.

**Fig. 19:**
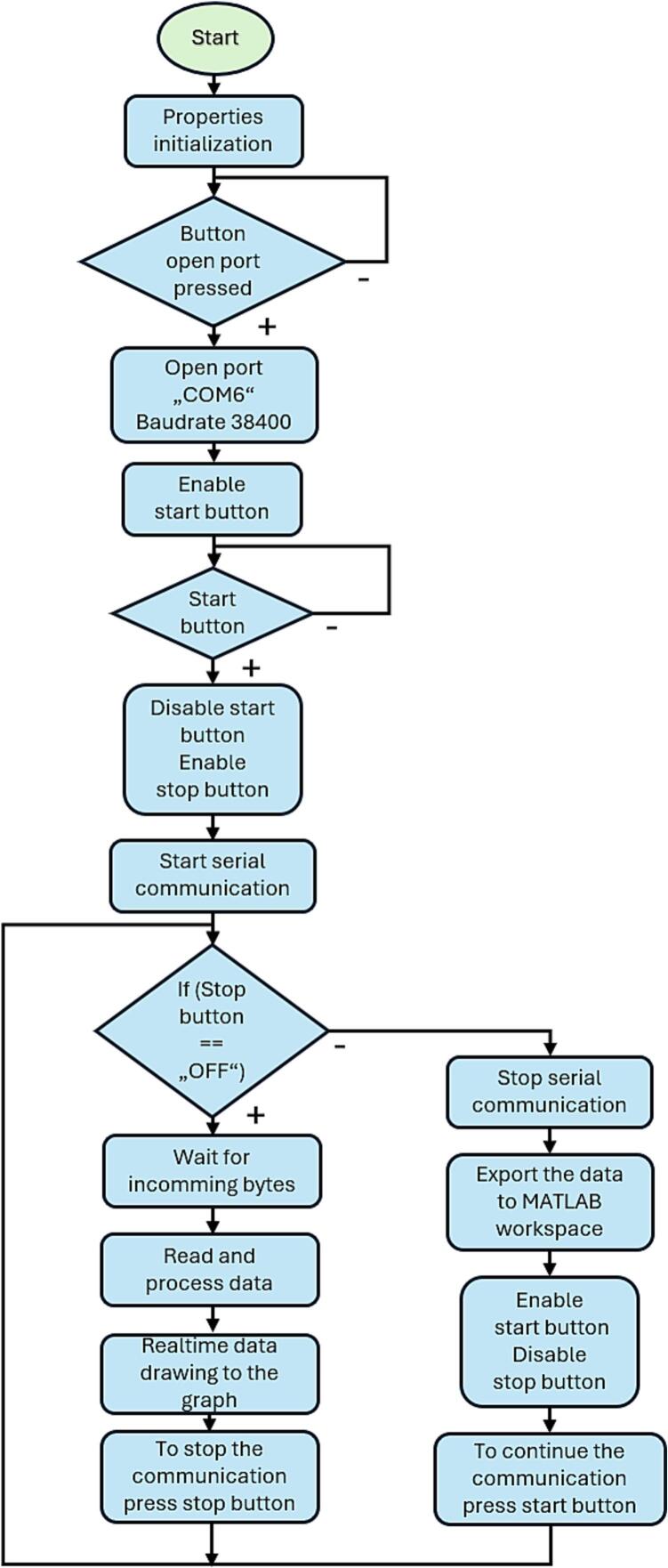
Designed MATLAB application flowchart.

After the communication setup is correctly configured, the application can be started. To initiate the measurement, click the 'OpenPort' button, Fig. 18. After clicking, wait for 5 s (establishing the communication), as repeated presses may cause errors. Once the connection is successfully established, the LED will change from blinking to a steady light. Measurement can then be started by clicking the 'Start' button. To stop the measurement, click the 'Stop' button. After closing the application, all raw data from 3-axis will be automatically sent to the MATLAB Workspace and can be found as data1, data2, and data3 Fig. 19..

### 6.1 Safety warnings and usage recommendations

During the development and use of the measuring device, it is essential to consider various safety aspects related to both the manufacturing process and its subsequent operation. When manually assembling and soldering components onto the printed circuit board, there is a risk of burns from contact with the heated soldering iron tip. It is recommended to use protective goggles and heat-resistant gloves, ensure a well-ventilated workspace, and avoid working near flammable materials.

During the 3D printing of the device enclosure, it is important to prevent contact with moving parts and hot surfaces (such as the heated bed). Adequate ventilation of the workspace must be ensured due to the potential release of fumes from plastic filaments.

Although the device operates at a low voltage (5 V), it is recommended to ensure proper power wiring, short-circuit protection, and insulation of the wires.

The device is not intended for use in humid, dusty, or chemically aggressive environments. Due to the potential for condensation or short circuits, it should only be operated in a dry, mildly temperature-controlled environment.

### 6.2 MATLAB application flowchart

For the possibility of modifying the application code and adapting it for personal use or research purposes, a detailed explanation of the individual code sections is essential.

After initializing the Magnetic Field Meter application, all graphical components of the user interface, such as individual graphs, are created. When the 'Open Port' button is pressed, the initialization of the graphs occurs, and the virtual serial interface (default speed is 38,400 Baud without any changes to the original code) on the defined communication port COM6 is created. After the initial setup, the 'Start' button becomes available. Pressing the 'Start' button activates serial communication and starts waiting for available data. At the same time, when the ‘Start’ button is pressed, the PC sends the character 'S' to the MCU via Bluetooth, thereby enabling serial communication. The application enters an infinite loop where it continuously monitors the number of available bytes in the buffer. If the byte count exceeds 450, 150 samples of type uint16 are read. The read data is divided into three graphs corresponding to the magnetic field axes X, Y, and Z in a 1:1:1 ratio. The samples are then visualized in real-time on the respective graph axes.

To stop the measurement, the 'Stop' button is made available, which halts data transmission from the sensor. At the same time, when the ‘Stop’ button is pressed, the PC sends the character 'X' to the MCU via Bluetooth, thereby disabling serial communication. All recorded samples for the respective period are sent to the MATLAB Workspace. The 'Stop' button is then disabled, and the 'Start' button is enabled.

If modification of the application is required, the sampling frequency and baudrate can be adjusted by simple changes in the code. Modifying the sampling frequency can be done easily using the variable Tvz, which is defined in the code as the sampling period, i.e., the inverse of the sampling frequency.

**Fig. 20:**
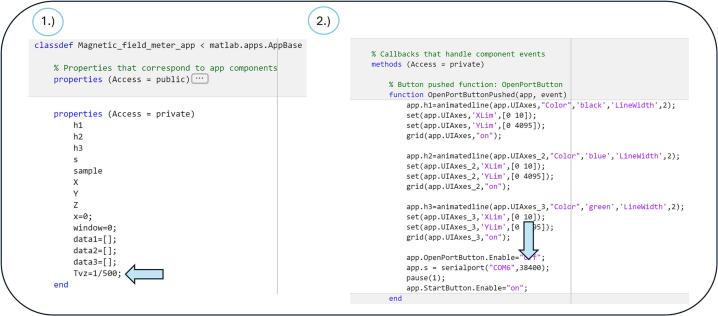
Modification of the application source code: 1) Sampling frequency adjustment. 2) Baudrate adjustment.

**Fig. 21:**
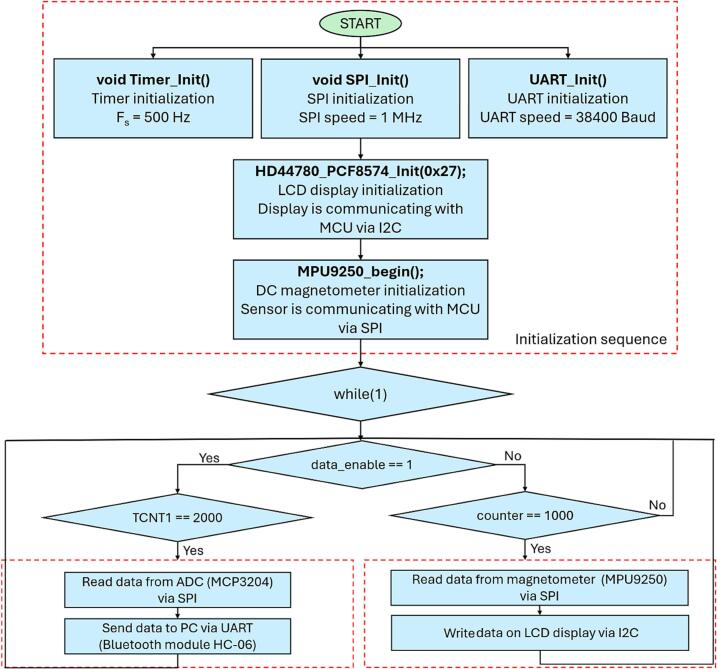
Microchip Studio firmware flowchart.

By changing Tvz = 1/500 or adjusting the denominator in the code, the sampling frequency can be modified (Fig. 20). Similarly, the baudrate can be adjusted by modifying the value in the following line of code: app.s = serialport(“COM6″, 38400); Fig. 21..

### 6.3 Microchip Studio firmware flowchart

For future modifications, it is important to understand the structure and function of the program in the Microchip Studio environment. During initialization, the SPI speed is set to the default 1 MHz, the baudrate to 38,400 Baud, and the sampling frequency to 500 Hz. The program runs in an infinite loop and continuously checks if serial communication is enabled. This is controlled by the variable data_enable, which is set to 1 when the MCU receives the byte (character) ‘S’ and set to 0 when it receives the byte ‘X’. This is managed by the MCU UART interrupt vector, which is activated upon receiving a byte from the PC via Bluetooth module. The program operates with two parallel branches. One branch executes every 2 ms, while the other runs every 2 s. The active branch is determined by the value of the variable data_enable. When data_enable is set to 0, data is read every two seconds via the SPI communication interface from the MPU9250 sensor, which measures the DC magnetic field. This corresponds to the condition counter == 1000 (1000 / Fs) × 1000  ms = 2 s, where Fs is sampling frequency for sending data to the PC. The obtained values for the X, Y, and Z axes are then displayed on an LCD screen via the I2C interface.

When data_enable is set to 1, the system instead transmits data (digitalized by ADC) from the MC858 sensor, which measures the AC magnetic field, to the computer via Bluetooth. In this case, the values for all three axes (X, Y, and Z) are sent continuously.

If it is necessary to change the sampling frequency, the value of the OCR1A register in the program must be adjusted according to the formula: *OCR1A* = sampling period / 1 µs. This allows precise control over the timing of data acquisition and communication processes.

The ATmega328P is a single-threaded processor and therefore does not support simultaneous display output and data transmission to the computer.

## 7 Validation and characterization

### 7.1 Adjusting the values of the HPF and LPF filters in the device

**Fig. 22:**
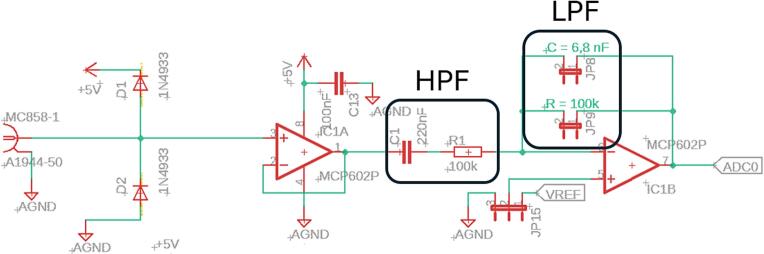
Realisation of HPF and LPF filters in schematic.

**Fig. 23:**
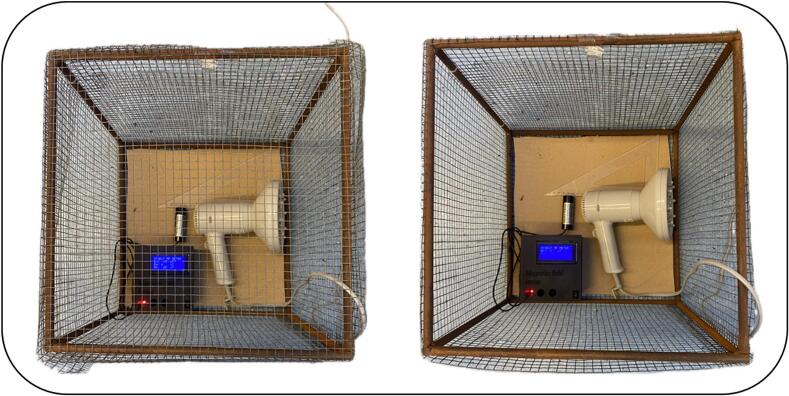
Visualization of the measurement setup: Faraday cage, hair dryer, and measuring device with sensor placed in Y position.

Given the possibility of customizing the measuring device - particularly in terms of adjusting the measurement range - it is essential to understand the relationships that enable the design and modification of analog circuits. To adjust the cutoff frequency of LPF filters located in the analog part of the device (Fig. 22), the following formula is required. While the LPF filters can be modified by replacing passive components, adjusting the HPF filters require physical modification of the PCB Fig. 23..

The cutoff frequency of the HPF and LPF filters is given by the following equation:

(1) $$F_{c}=\frac{1}{2∙\pi ∙R_{1}∙C_{1}}$$

In the case of a given desired cutoff frequency F_c_​ (Hz), it is possible to calculate appropriate values for the components R_1_​ (Ω) and C_1_​ (F). A practical approach is to select one of these values based on component availability and then calculate the other. If the value of resistor R_1_​ is known, the capacitance can be determined using the following formula:

(2) $$C_{1}=\frac{1}{2∙\pi ∙F_{c}∙R_{1}}$$

It should be noted that adjusting the values of passive components in the filter may also affect the gain of the entire analog circuit. The gain G of the inverting OA is defined by the following equation:

(3) $$G=-\frac{R}{R_{1}}$$

### 7.2 Calculation of the magnetic field flux density from the measured data

After finalizing the functionality verification of the device, experimental measurements of the magnetic field can be carried out. Given that the sensor output is in millivolts (mV), it is necessary to perform calculations that allow for obtaining the final values of the magnetic flux density ***B*** of the magnetic field.

Each MC858 sensor undergoes a NIST traceable calibration guaranteed by manufacturer to ensure accuracy: ±4 % typical calibration tolerance (±5 % worst case) from 10 Hz to 400 Hz. Calibrated to ANSI Standard 644–1987 [12]. The corresponding calibration curve of the sensor is also presented in publication [3], providing additional validation of its metrological performance. The frequency response curve of MC858 can be found at the manufacturer's website [24].

This study presents a PCB designed with an active bandpass filter, an ADC, and a microcontroller to capture, process, and transmit data to PC. The signals are detected by three magnetic coils connected to the PCB.

The signals come from devices on power lines, where the current generates a time-varying magnetic field ***B*** (T). According to Faraday’s law, this magnetic field induces an electric field ***E*** (V/m):

(4) $$\nabla \times E=-\frac{\partial B}{\partial t}$$

Moreover, Ampère’s Law, including Maxwell’s correction, establishes the relationship between the magnetic field ***H*** (A/m), the electric displacement field ***D*** (Aּs/m^2^), and the current density ***J*** (A/m^2^):

(5) $$\nabla \times H=J+\frac{\partial D}{\partial t}$$

The magnetic coils detect changes in the magnetic field by measuring the voltage induced within them. For a coil consisting of N turns and covering a surface area A (m^2^), with magnetic flux trough the coil $\Phi_{B}$ (Wb), the induced electromotive force ε (V) is calculated as:

(6) $$\varepsilon =-N\frac{d\Phi_{B}}{\mathrm{dt}}$$

At the same time, under conditions of ideal geometrical arrangement and perfect orthogonality, the mutual inductance between any two coils in a triaxial magnetic field sensor is negligible, approaching zero due to the absence of magnetic flux linkage between perpendicularly oriented coil axes [25,26,27].

The induced voltage is then processed by the PCB. In the proposed solution, a 12-bit ADC (MCP3204) is used, with a resolution of 2^12^ = 4096 discrete levels. The quantization step *q* of the used ADC can be calculated using the following formula:

(7) $$q=\frac{5000\;mV}{4096-1}=1.221\;mV$$

It follows that a change of one digital level corresponds to a change in the output voltage of approximately 1.221 mV. Based on the sensor's technical documentation, the sensitivity is defined as 22 mV/μT, which means that a change in magnetic flux density of 1 μT will cause a change in the sensor’s output induced voltage of 22 mV. The relationship between one digital level and magnetic flux density ***B*** (µT) is then:

(8) $$B=\frac{1.221\;mV}{22\;mV/\mu T}=0.0555\;\mu T$$

It follows that one quantization step of the A/D converter corresponds to 0.0555 μT. For practical calculation of magnetic flux density ***B*** (µT) values from the sensor’s output induced voltage, the modified formula can be used:

(9) $$B=\frac{U_{OUT}}{22\;mV/\mu T\;}$$

*U*_OUT_ (V) represents RMS value of the sensor’s output induced voltage. For simplification of the conversion, a helper script (Magnetic_induction_MC858.m) was created in the MATLAB environment, which allows for quick calculation of the magnetic flux density from the input voltage for any axis of the sensor.

### 7.3 Test measurements

For the purpose of verifying the functionality of the proposed measurement system, a series of experimental measurements were conducted. The measurements took place in a Faraday cage specifically designed to shield electromagnetic interference in the ELF range (≤300 Hz). The cage was constructed from a steel mesh with a mesh size of 1 cm. This cage design ensures sufficient shielding efficiency in the low frequency range, while still allowing wireless communication via Bluetooth between the measuring device and the computer. The sensors were connected via coaxial cables to BNC connectors 1, 2, and 3.

**Fig. 24:**
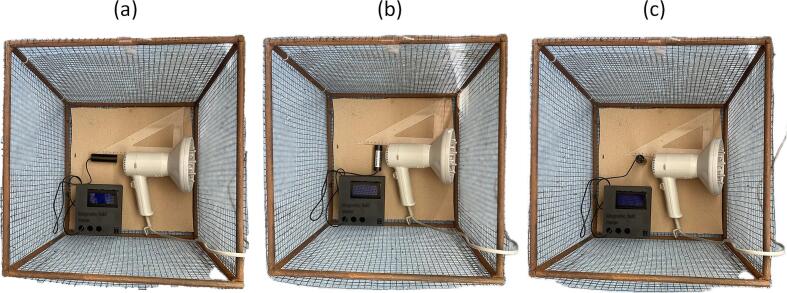
Orientation of the sensor relative to the power electronics integrated in the hairdryer: (a) X-axis, (b) Y-axis and (c) Z-axis.

Inside the Faraday cage, an electrical device commonly used in domestic environments (hair dryer) was placed, serving as the source of the electromagnetic field. The device was positioned at distances of 1 cm and 3 cm from the MC858 sensors, ensuring direct exposure of the sensors to the field generated by the device's power electronics along the X, Y, and Z axes, Fig. 24.

The device was configured with the above-mentioned values for the HPF and LPF filters. The DC offset (voltage shift) of the OA was set to 2.5 V, the baudrate was set to 38,400 Baud, and the sampling frequency was set to 500 Hz.

For each distance, four phases of measurements were performed with five repetitions. The first phase of measurement took place in a closed Faraday cage without any electrical device. In the second phase of measurement, the electrical device (hair dryer) was connected to the system, but not activated. The last two phases of measurements were carried out with the hair dryer turned on – first at the low power setting and then at the high-power setting. The duration of each measurement with hair dryer on was 20 s, with the hair dryer being turned on 10 s before the start of the recording to stabilize the measurement conditions. Each measurement was repeated five times to verify the repeatability and reliability of the measured data. The current flowing through the wire to the hair dryer at low power setting was measured as 0.172 A. At high-power setting these values were 0.320 A. The measurements were conducted using the YATO YT-73091 [28] digital clamp meter (YATO, Wroclaw, Poland).

**Fig. 25:**
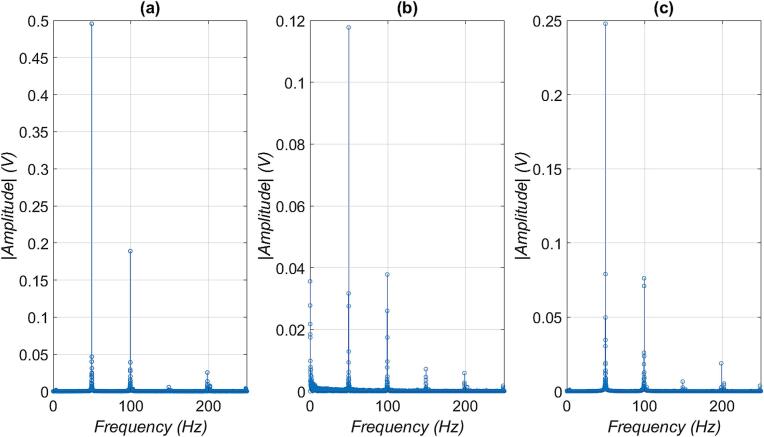
Fast Fourier Transform of measured signals, hair dryer was set to high power mode: (a) X-axis, (b) Y-axis, and (c) Z-axis.

A frequency analysis was performed on the measured data using the Fast Fourier Transform (FFT), which allows the transformation of the time-domain signal into the frequency domain. The results of this analysis are shown in the Fig. 25. for the individual X, Y, and Z axes. In all three cases, the dominant frequency component at 50 Hz can be observed, which corresponds to the fundamental harmonic frequency of the mains electricity used in EU.

In the analysis of the signal along the X-axis, the dominant frequency component at 50 Hz had an amplitude of approximately 0.49 V, with higher harmonics clearly visible, particularly the second harmonic at 100 Hz and the fourth at 200 Hz.

The Y-axis shows a similar spectral characteristic, but with lower amplitude of the main component – approximately 0.12 V at 50 Hz. Harmonic frequencies (100 Hz, 150 Hz, 200 Hz) are also present.

The FFT analysis of the signal on the Z-axis reveals an intermediate amplitude of the dominant component at 50 Hz, reaching approximately 0.25 V with visible harmonics at 100 Hz, 150 Hz and 200 Hz.

These differences in amplitude across the axes can be attributed to the spatial orientation of the sensor relative to the internal power electronics of the hair dryer. The X-axis exhibits the highest amplitude due to its alignment parallel to the power components, resulting in stronger coupling with the generated electromagnetic field. In contrast, the Y-axis shows the lowest amplitude, likely because it is oriented perpendicularly to the power components in hair dryer. The Z-axis displays intermediate amplitude values, corresponding to a vertical positioning of the sensor, which results in partial alignment with the field distribution.

**Fig. 26:**
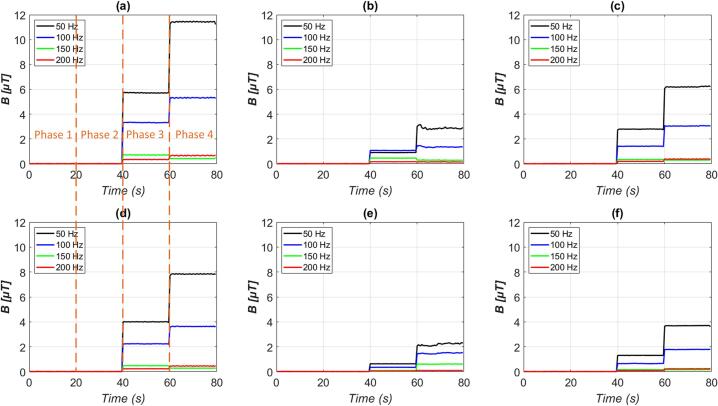
Magnetic field induction calculated from the signal of a hair dryer along the X, Y, and Z axes at distances of 1 cm and 3 cm: (a) X-axis at 1 cm, (b) Y-axis at 1 cm, (c) Z-axis at 1 cm, (d) X-axis at 3 cm, (e) Y-axis at 3 cm, and (f) Z-axis at 3 cm.

The Fig. 26. illustrates the temporal development of magnetic field induction across the X, Y, and Z axes, measured at distances of 1 cm and 3 cm from a common household electrical device (hair dryer) under four operational phases. The device acts as a source of electromagnetic disturbance in the ELF band.

During Phase 1, when the hair dryer is disconnected from the power grid, the recorded magnetic field induction remains close to zero across all axes and frequencies. This phase represents the baseline noise level in the Faraday cage, confirming the effectiveness of electromagnetic shielding.

In Phase 2, the device is connected to the mains power supply but remains switched off. Here also the measured magnetic field induction remains close to zero across all axes.

Significant changes occur in Phase 3, where the hair dryer is powered on at its first operational level, current to the dryer is approx. 0.172 A. Here, a pronounced increase in the 50 Hz component of the magnetic induction is observed across all axes, with the most significant rise occurring along the X and Z axes. Additionally, the presence of higher harmonics (notably 100 Hz and 150 Hz) becomes more prominent.

In Phase 4, when the hair dryer operates at full power (second level, current to the dryer is approx. 0.320 A), the amplitude of the magnetic induction increases further, especially at 1 cm, where the B-field at 50 Hz approaches 11–12 μT on the X-axis. This phase represents the maximum electromagnetic emission scenario, with a clear escalation of both the fundamental frequency and its harmonics. The magnitude is notably reduced at 3 cm, indicating the spatial attenuation of the magnetic field with distance. The measured values of the DC magnetic field were −16 µT in the X-axis, 26 µT in the Y-axis, and −17 µT in the Z-axis.

Overall, the data clearly demonstrate a direct correlation between the operating state of the device and the intensity of the magnetic field.

Measuring DC and ELF fields is important for health, safety, and scientific reasons. ELF fields, from power lines and appliances, may impact health. DC fields, like Earth’s magnetic field or those from trains, can affect sensitive devices. These measurements help detect faults, prevent interference, support research, and ensure compliance with safety standards.

## Ethics statements

The authors have nothing to declare under this heading.

## CRediT authorship contribution statement

**Veronika Wohlmuthova:** Writing – original draft, Visualization, Validation, Software, Methodology, Investigation, Formal analysis, Data curation, Conceptualization. **Michal Labuda:** Writing – review & editing, Validation, Supervision, Software, Methodology, Funding acquisition, Formal analysis, Data curation. **Mariana Benova:** Writing – review & editing, Supervision.

## Declaration of competing interest

The authors declare that they have no known competing financial interests or personal relationships that could have appeared to influence the work reported in this paper.
